# Neuro-Ophthalmologic Complications of SARS-Cov-2 Infections and Vaccinations

**DOI:** 10.18502/jovr.v19i1.15451

**Published:** 2024-03-14

**Authors:** Josef Finsterer, Fulvio Alexandre Scorza, Ana Claudia Fiorini

**Affiliations:** ^1^Neurology and Neurophysiology Center, Postfach 20, Vienna, Austria; ^2^Disciplina de Neurociência, Universidade Federal de São Paulo/Escola Paulista de Medicina (UNIFESP/EPM), São Paulo, Brazil; ^3^Programa de Estudos Pós-Graduado em Fonoaudiologia, Pontifícia Universidade Católica de São Paulo (PUC-SP), Brazil; ^4^Departamento de Fonoaudiologia, Escola Paulista de Medicina/Universidade Federal de São Paulo (EPM/UNIFESP), São Paulo, Brazil

## Dear Editor,

We read with interest the narrative review by Feizi et al about the neuro-ophthalmologic complications of SARS-CoV-2 infections and vaccinations.^[1]^ It was concluded that SARS-CoV-2 infections and all SARS-CoV-2 vaccines can affect the afferent and efferent visual pathways due to papillophlebitis, idiopathic intracranial, hypertension, optic neuritis, ischemic stroke, pupillomotor pathway impairment, and cranial nerve dysfunction.^[1]^ The study is comprehensive, but has limitations that are a cause for concern and should be discussed.

Headaches should not always be classified as the neuro-ophthalmologic manifestation of SARS-CoV-2 infections or vaccinations. Headache, though regarded as one of the most common complications of SARS-CoV-2 infections and vaccinations, rarely affects the eyes primarily. Only if infection or vaccination induces new onset migraine with visual aura^[2]^ or flare-ups of migraine with visual aura, if vaccination triggers status migraenosus as reported as a complication after vaccination with the BPV,^[3]^ or if headache occurs associated with eye-pain, this can be considered as neuro-ophthalmologic manifestation.

In addition to migraine with visual aura, several other neuro-ophthalmic disorders were not discussed in the review. The Vogt-Koyanagi-Harada syndrome, which has been repeatedly reported in patients after SARS-CoV-2 vaccination^[4]^ was not addressed. There are also reports of individual patients developing central retinal artery occlusion after vaccination with an mRNA-based SARS-CoV-2 vaccine.^[5]^ There are also several reports of patients suffering giant cell arteritis (GCA) involving retinal arteries after SARS-CoV-2 vaccination.^[6]^ In a female with reversible, cerebral vasoconstriction syndrome (RCVS), bilateral scotomas were the first symptoms along with thunderclap headache 18 days after the Moderna vaccine.^[7]^ Multiple evanescent white dot syndrome (MEWDS) after SARS-CoV-2 infection is also not mentioned.^[8]^ MEWDS is characterized by painless visual loss and typical retinal changes. Also not discussed was sicca syndrome due to impaired innervation of the lacrimal glands following SARS-CoV-2 vaccination.^[9]^ Central retinal vein occlusion after BPV was also not addressed.^[10]^ Another rare complication of SARS-CoV-2 vaccinations that worries the neuro-ophthalmologist is acute retinal necrosis (ARN).^[11]^ Only four cases with this uncommon, devastating cause of vision-threatening uveitis have thus far been reported.^[11]^ In addition to arteritic anterior ischemic optic neuropathy (AAION), non-arteritic anterior ischemic optic neuropathy (NIAION) has been reported as a complication of SARS-CoV-2 infection.^[12]^ Furthermore, there is no mention of autoimmune encephalitis after SARS-CoV-2 vaccination, which can manifest itself with incoordination, lethargy, gait disturbance, and visual disturbances. Since vaccine-induced thrombotic thrombocytopenia (VITT) can be complicated not only by thrombosis but also by intracerebral bleeding, and bleeding can lead to visual disturbances, it must also be included in the discussion. Last but not least, the pituitary apoplexy has been described as a complication of SARS-CoV-2 infection and can manifest itself with bitemporal hemianopia.

Overall, in our opinion, this interesting study has limitations that may call the results and their interpretation into question. Addressing these issues would strengthen the conclusions and could improve the status of the study. The spectrum of neuro-ophthalmologic impairments after a SARS-CoV-2 infection or vaccination is broader than what was discussed in the review article. In order to be able to assess the consequences of a SARS-CoV-2 infection or vaccination on the post-COVID/long-COVID-syndrome and the long post-SARS-CoV-2 vaccination syndrome, it is crucial to look at the entire spectrum encountered so far.

##  Financial Support and Sponsorship

None.

##  Conflicts of Interest

None.
